# Barriers for the inclusion of sexuality in nursing care for women with gynecological and breast cancer: perspective of professionals^1^

**DOI:** 10.1590/0104-1169.3602.2528

**Published:** 2015

**Authors:** Simone Mara de Araújo Ferreira, Thais de Oliveira Gozzo, Marislei Sanches Panobianco, Manoel Antônio dos Santos, Ana Maria de Almeida

**Affiliations:** 2 Doctoral student, Escola de Enfermagem de Ribeirão Preto, Universidade de São Paulo, WHO Collaborating Centre for Nursing Research Development, Ribeirão Preto, SP, Brazil; 3 PhD, Professor, Escola de Enfermagem de Ribeirão Preto, Universidade de São Paulo, WHO Collaborating Centre for Nursing Research Development, Ribeirão Preto, SP, Brazil; 4 PhD, Associate Professor, Faculdade de Filosofia, Ciências e Letras de Ribeirão Preto, Universidade de São Paulo, Ribeirão Preto, SP, Brazil; 5 PhD, Associate Professor, Escola de Enfermagem de Ribeirão Preto, Universidade de São Paulo, WHO Collaborating Centre for Nursing Research Development, Ribeirão Preto, SP, Brazil

**Keywords:** Nursing, Sexuality, Genital Neoplasms, Female, Breast Cancer, Comprehensive Health Care

## Abstract

**AIM:**

qualitative study, which aimed to identify the barriers that influence nursing care practices related to the sexuality of women with gynecological and breast cancer.

**METHODS:**

the study was conducted with 16 professionals of the nursing area (nurses, nursing technicians and nursing assistants) from two sectors of a university hospital situated in the state of São Paulo, Brazil. The data was collected using semi-structured, in-depth individual interviews. All the interviews were recorded and the participants' responses were identified and categorized using Content Analysis.

**RESULTS:**

three major themes were identified. These are as follows: 1) barriers related to the biomedical model; 2) barriers related to institutional dynamics and 3) barriers related to the social interpretations of sexuality.

**CONCLUSIONS:**

the results of this study showed that the systematized inclusion of this issue in nursing care routines requires changes in the health paradigm and in the work dynamic, as well as reflection on the personal values and social interpretations related to the topic. A major challenge is to divest sexuality of the taboos and prejudices which accompany it, as well as to contribute to the nursing team being more aware of the difficulties faced by women with gynaecological and breast cancer.

## Introduction

Gynecological and breast cancer not only represent public health problems, but also affect organs to which important symbolic values are attributed in the Brazilian culture, mainly related to the area of sexuality and body image. The disease itself, along with its treatment, frequently entails significant and long-lasting poor sexual health^(^ 1 ^-^ 4 ^)^, with changes in relationships - principally in the relationship with the sexual partner - occurring during and after the treatment^(^ 5 ^-^ 12 ^)^.

In spite of the fact that the sexuality of the women affected is compromised, health professionals often fail to adequately assess such problems, or even address them at all. It is rare for there to be open discussion and exploration of alternatives - not limited to the sexual relationship - for promoting the expression of affective-sexual intimacy between the woman and her partner^(^ 5 ^,^ 13 ^-^ 14 ^)^. Even recognizing that the assessment of sexuality is an intrinsic part of the care, for various reasons, many nurses do not address these questions in care practice^(^ 5 ^,^ 13 ^,^ 15 ^-^ 16 ^)^.

Although studies highlight the difficulties in communication related to the subject of sexuality in the practice of the oncology nurse, gaps remain in the knowledge regarding the barriers which impede the topic's inclusion in the nursing care. The relationship between oncology nurses' attitudes and perceptions regarding sexuality in care practice must be explored, in order to possibly understand why they so often fail to conclude an assessment of the patient's sexual health during the care^(^ 5 ^)^. It should be noted that in the institution where the present study was undertaken there is no formal sexual counselling service, which becomes more serious when considering that the body occupies a central position in Brazilian culture, principally among women. There is a particular body model held in high regard, and the continuous preoccupation with obtaining a perfect body is notorious^(^ 17 ^)^.

A broader focus on this problem is needed, so that the experiences and meanings of these women can be valued and considered in nursing care practices. This is because the concept of sexuality cannot be separated from health, as sexuality and intimacy-related issues are fundamental to maintaining the well-being and self-esteem of women affected by cancer^(^ 6 ^,^ 18 ^)^. Considering that sexuality cannot be neglected in care, which barriers hinder the inclusion of the topic in nursing care? This study aimed to identify the barriers which influence nursing care practices related to the sexuality of women with gynecological and breast cancer.

## Methods

As the study addresses the universe of meanings, motives, aspirations, beliefs, values and attitudes of Brazilian nursing professionals, a qualitative approach was selected.

### Participants, setting and sample

The sample was selected by convenience, in which the informants were chosen to meet the objective of the study. It should be noted that in Brazil, nursing work is undertaken by different categories of workers. These professional categories (nursing assistant, nursing technician and nurse) have distinct training processes and distinct sets of professional activities and responsibilities. The Nurse performs all nursing activities, the Nursing Technician performs mid-level activities, involving the guidance and monitoring of nursing work in a support role, and participation in the planning of the nursing care. The Nursing Assistant performs mid-level activities of a repetitive nature involving nursing support services under supervision, as well as participates in the execution of simple treatment processes^(^ 19 ^)^. The professional functions of the nursing assistant are characterized by a set of technical procedures^(^ 20 ^)^. It is the responsibility of the nurse to exercise the functions of organizing and managing the service, coordinating the actions of the nursing team professionals.

Considering the functions of each category, 16 nursing professionals, all female, were included, these being four nurses, three nursing technicians, and nine nursing assistants. Of this total, nine worked on the ward (the Nursing Section in the Gynecology Centre) and seven in the outpatient unit (Mastology and Gynecological Oncology Outpatient Centre) of a university hospital situated in the state of São Paulo, Brazil. The inclusion of professionals from two units broadened the perspective of the assessment, as this highlighted distinct care contexts for the same type of patient.

### Ethical considerations

The study was approved by the Research Ethics Committee of the University of São Paulo at Ribeirão Preto College of Nursing. Following the approval, contact was made with the nurses responsible for the two fields of investigation, so as to gain their participation in the undertaking of the study. Next, each nursing professional selected as a source of information was individually invited to participate and the aims of the study were explained to them. The terms of consent were explained and signed by all participants.

To preserve the identities of the nursing professionals, an alpha-numerical system was chosen, with the codename 'I' (for 'Interviewee') followed by an Arabic numeral, according to the order of performance of the interviews (I1, I2, I3... I16). The professional category was stated (nurse, nursing technician, or nursing assistant), as well as where each professional worked (Ward or Outpatients).

### The interview process

Data were collected between May and July 2011. The first author conducted semi-structured, in-depth individual interviews. The interviews were previously scheduled and held in a private room in the workplaces of the nursing professionals. The average duration of each interview was 30 minutes. The interviews were recorded on a digital recorder. The basic questions were listed on a script and covered issues related to the care practice, inclusion in the profession, specific questions on sexuality, and experiences related to the issue in the care context.

### Data analysis

Data analysis was not carried out at a pre-determined time, with the performance of the interviews and the analysis of the data occurring simultaneously, from the start of the data collection process through to the end. The recordings of the interviews were downloaded to a computer, using the Voice Editing program, version 1.0, after which they were transcribed in full. Aiming to preserve the original narratives, only a few adaptations to standardized language were made, attempting to maintain the characteristics of individual expressions and the mistakes made by the interviewees in their use of language.

To identify the barriers which influence nursing practices related to sexuality and care for women with gynecological and breast cancer, the aim was to understand the cultural context in which this care is produced, starting from the premise that this context determines the actions and perceptions of nursing professionals in relation to this issue. Thus, the professional work spaces and the interactions established by the subjects involved in this process were taken into account.

Once this backdrop for supporting the analysis had been developed, the material itself was explored. To guide the work, Content Analysis methodology^(^ 21 ^)^ was adopted. Through specialized and scientific procedures, this methodology allows the replication and validation of inferences from data extracted from a specified context - in this case, the hospital environment. This process uses inductive reasoning, through which themes and categories emerge from the data by means of thorough examination and constant comparisons. This analysis cannot be separated from deductive reasoning, as concepts and variables from previous studies and other theories are also used, especially at the beginning of the analysis.

After reading the material from the interview several times, phrases were selected as the units of analysis, guided by the following themes: Prioritizing technical aspects; Focusing on controlling the disease and the physical complications originating from the treatment; Turnover within work sectors; Lack of time; Excessive institutional bureaucracy; Lack of private areas; Sexuality being situated in the sphere of prohibitions; Gender issues; and Patients often feeling guilty and ashamed to think about sex. The cultural influences which guide nursing practice, and the incorporation of sexuality into the care, were considered in the interpretation of the data^(^ 22 ^)^.

The barriers mentioned by the nursing professionals as obstacles to addressing sexuality in care practice were grouped into three categories: *Barriers related to the health model*; *Barriers related to the institutional dynamics* and *Barriers related to the social interpretations of sexuality*.

## Results and Discussion

### Barriers related to the health model

The health institution where the study was developed adopts care practices based on the biomedical model. In this context, the nursing team reproduces the same precepts, which according to the reports of the professionals results in a standardized and biological technical health practice. In addition to reproducing the current health model, administrative precepts are incorporated to organize the nursing work process. Thus, the accounts of the professionals prioritize technical aspects, valorizing care for treating the physical body and putting too much emphasis on the organization of the environment where the work is performed, as observed in the reports. *We arrive, first we go through the wards, check the patients' vital signs, see how they are doing, then listen to the main pain complaints, and see what the priority is. There's a drip finishing, get a summary from each ward and for each patient to be able to monitor them during the rest of the shift. Check the vital signs, administer the medication. (I8, nursing assistant, Ward)*

When prioritizing actions and practices, the team considers it essential to maintain life; the nursing professionals do not recognize that other care aspects, involving issues of women's subjectivity, are as important as care for the physical body. *I've already talked to some [patients] don't worry about your hair, or about your breast, right, first worry about preserving your health. (I11, nursing assistant, Outpatient clinic)*

Because nursing reproduces the biomedical model, it defines the priority actions to be implemented with women with gynecological and breast cancer. Professionals are concerned with meeting what they call the basic needs, however, this is restricted to carrying out practices aimed at controlling the disease and the physical complications due to the treatment. Considering the attitudes of cancer care nursing professionals, all of them are focused on the cancer treatment, disregarding sexuality and its importance for the wellbeing of the patient^(^ 7 ^)^.

Focusing on the cure, and based on the delivery of a technical view of care, nursing fails to value the subjective aspects of care, and denies the holistic nature of the subjects. Similar characteristics were found in a study undertaken with nurses in six hospitals in China. Although healthcare in that country is changing, the current practice still follows the biomedical model. Thus, Chinese nurses were not autonomous and felt insecure to address subjective issues, such as sexuality in care delivery to gynecological cancer patients^(^ 23 ^)^.

### Barriers related to the institutional dynamics

The organizational culture of the institution contributes to the nursing team neglecting to address sexuality. Bonding difficulties develop from the work organization, which represents a decisive barrier to addressing sexuality with women undergoing cancer treatment. This difficulty is partially perceived by the nursing professionals, who explain its existence by indicating the staff turnover within the work sectors as one of the aspects that interferes negatively in bonding with the patients. The professionals interviewed frequently alternate between sectors, shifts and even workplaces, this being the situation on the wards as well as at the outpatient clinic.

I change, change. Like, last month I was in wound dressing, the month before I was in orientation, now I'm in the screening service. Then, when I work afternoons, I sometimes go to the materials section. (I6, nursing technician, Outpatient clinic)

Here, in my case, I don't work a fixed shift pattern. I work mornings, afternoons and nights. (I8, nursing assistant, Ward)

Another characteristic of the institutional dynamics that makes it difficult to address the issue, which was evidenced in the statements, is the lack of time, which was identified when the professionals classified the care delivered as rapid - which makes addressing the issue of sexuality impossible.

I don't know if it's because everything here goes so fast, you see, you've got a lot of patients to take care of. How are you supposed to have time to address that, if sometimes you can't even see all the patients and do, let's say, progress reports for them, you see? (I14, nurse, Ward)

The institution provides care for a large number of patients, with a limited number of staff to perform this complex task. Contacts were characterized as fast and automatic. The nursing professionals were often unable to sit and talk to the patients during their shift and, considering the amount of information to be transmitted, sexuality was not given priority as an issue during care.

When the shift is calmer, you sit down, talk, listen to stories, various stories, even like, to talk about things that are worrying them in relation to intimacy with the husband, in relation to their child, their life... (I1, nursing assistant, Ward)

Another aspect reinforced by the professionals interviewed was the excessive institutional bureaucracy, which makes it impossible for the professionals and patients to have a productive dialogue. There are so many forms to complete and so much guidance to provide that the work becomes mechanical and carried out by rote. Therefore, there is no time left for women undergoing cancer treatment to present their doubts.

The routine, paperwork, lots of forms, you complete a lot. The patient arrives here for us with a lot of paper because we have to inform her about everything. So, we hardly even ask her about herself. There are so many small items required that we don't have enough time with the patient. (I3, nursing assistant, Outpatient clinic)

Remaining with the issue of the institutional dynamics, the physical space also contributes to the issues not appearing during nursing actions. The team highlights the lack of private areas as an obstacle to patients being able to freely air their concerns.

To provide guidance for example... Giving guidance more privately, in a place like this [private]. Because sometimes, she also has a doubt about something, but she won't tell you. We give guidance on the ward with five others. (I10, nursing technician, Ward)

It can be seen that the dimension of sexuality is understood as an aspect that demands greater contact with professionals if it is to be expressed comfortably. Thus, the work dynamics at the institution do not allow women undergoing cancer treatment to be sufficiently close to the team to express their subjective needs. Difficulties in establishing bonds derive from a lack of time, as well as the high turnover rate of professionals within the different sectors. The need to rely on good bonds to allow sexuality-related issues to emerge in health practices was also reported in another study^(^ 16 ^)^. In this study, the comfort levels Turkish nurses experienced during clinical experiences were assessed, which included sexuality topics. Nurses from clinical units felt more comfortable than nurses working with surgical patients. The author explained this finding by suggesting that, probably, long periods of hospitalization favor addressing these topics, strengthening the intimacy between professionals and patients, which is frequently the case in clinical units. More than 80% of the nurses who participated in a study undertaken in China agreed that having a good relationship with the patient and being able to communicate facilitate the inclusion of the issue of sexuality in nursing practices^(^ 23 ^)^.

The vulnerability of the nursing professionals is provoked by their physical closeness and involvement with the women with gynecological and breast cancer, leading them to construct barriers to avoid becoming closer and bonding with the service users. Reflecting on experiences of pleasure and suffering in nursing team work, one study^(^ 24 ^)^ mentioned that the work of the team creates ambiguous feelings, which can contribute to experiences of pleasure as well as suffering. If, from one perspective, workers feel useful when serving and comforting, from another, they suffer when they are confronted with the imminence of death and difficult situations permeated with pain and suffering.

The lack of time allocated for the care which encompasses the sexuality of these women, can result from the excessive number of patients attended, the limited number of staff and the countless and often bureaucratic activities to be performed. In a study undertaken in Greece^(^ 15 ^)^ nurses also indicated a lack of time and heavy workload as external factors that make it difficult to discuss sexual problems with patients.

In a study regarding personal attitudes and beliefs about incorporating a patient sexuality assessment and counseling into nursing practice, 148 nurses were interviewed, who worked in selected inpatient units and outpatient clinics from a large metropolitan medical centre in Detroit (USA). An important result highlighted that the majority of the nurses (70.2%) reported that there was no time to discuss sexuality with their patients^(^ 25 ^)^. Similar characteristics were also reported in the hospital context in China. Chinese nurses believed that insufficient staff - resulting in a lack of time and energy - and insufficient resources were the main barriers to addressing sexuality^(^ 23 ^)^.

The results of a study^(^ 26 ^)^ that explored the sexuality-related experiences of 52 patients during cancer treatment showed some reasons perceived by the patients for sexuality not being considered in the nursing care. One of the aspects highlighted was the assertion that nurses are too busy to sit down and talk about these issues with the patients. In addition to the lack of time, the organization of institutional spaces did not provide places where dialogues could take place in private. This can represent an important barrier for women with gynecological and breast cancer to express their sexuality, given that cultural values still permeate the issue and place it in the private sphere. Privacy makes it possible to address sexuality, as it creates a favorable climate for confidentiality^(^ 7 ^)^. Chinese nurses were also aware of the need to offer an environment that favors having these discussions with the patients^(^ 23 ^)^.

### Barriers related to the social interpretations of sexuality

From the sociocultural perspective, sexuality is situated in the sphere of prohibitions and, thus, addressing it is often only touched upon or even avoided. The professionals interviewed considered sexuality a sensitive and delicate issue which belongs in the private sphere, therefore many women do not feel comfortable talking about intimate matters in the hospital environment. This repression is intensified when sexuality is considered from the perspective of gender. Inequalities in the construction of the male and female roles characterize life in society. This topic is considered inappropriate because, throughout their socialization process, women are subject to continuous repression in the organization and expression of their sexuality.

I think it's really because of this gender issue, because women end up being very withdrawn and not talking. I think it's because it's such an intimate thing, right? So, it's a very restricted thing, right, which we learn since when we are very young, you know, that there are some things restricted to your home, which you don't talk about, right? (I13, Nurse, Ward)

The nursing professionals also explained the absence of addressing sexuality in nursing care as being due to the women undergoing cancer treatment reproducing, in the hospital environment, the cultural issues they experience in the process of constructing their female identity. Due to current gender standards, women learn to isolate themselves early, therefore they do not raise the issue with the team. Corroborating this perception of the professionals, the results of a study involving 202 Chinese nurses showed that 77.7% of them saw sexuality as an issue which is too private to discuss with the patient and 63.4% assumed that most hospitalized patients are too ill to take interest in sexuality related issues^(^ 23 ^)^. Similar results were found by a study conducted in a large metropolitan medical center in the USA with nurses, which showed that they believed sexuality to be a private matter which, therefore, should not be addressed. They were more inclined to believe that hospitalized patients were too ill to be interested in sexual issues^(^ 25 ^)^.

This prejudice involved in the theme makes it difficult to include it in healthcare actions. In addition to the embarrassment of the patients, there is the discomfort of the health team. Hence, it cannot be ignored that nursing, as a predominantly female profession, transports the standards and values inherent in the cultural construction of women's sexuality into the profession. The gender issues that influence the construction of women's sexuality are the same ones that determine nursing actions and prevent them from expressing their sensitivity, leading to the censorship of discussions related to the topic and further increasing prejudice. Nurses often feel too uncomfortable and embarrassed to discuss sexuality with the patients and are afraid of violating their privacy^(^ 13 ^,^ 23 ^)^.

Regarding privacy and comfort, the majority of the nurses (81.7%) did not believe that sexuality was an issue too private to discuss; however, nearly half of the nurses (47.9%) felt uncomfortable talking about sexual issues^(^ 25 ^)^.

These attitudes of the nursing team are related to professional education, with cultural influences originating from values, meanings, symbols and specific concepts. Nurses have learned the basic rule that care delivery is natural, serious and without embarrassment. Difficulties in interacting with the sexuality of others are associated with the suppression of sexuality in the profession and with the adoption of behavioral standards through morality rather than what is considered to promote health^(^ 27 ^)^. According to the authors, nurses working with cancer patients have difficulty in exploring the issue and tend to avoid it, leaving patients with many unanswered questions.

Nurses who believe that sexuality is a topic too private to discuss with their patients also feel that sexual issues should only be addressed if and when patients initiate the conversation. These same nurses usually postpone these discussions and hope that another professional, whom they consider better trained to address this issue, might take the initiative and offer patients advice^(^ 5 ^)^.

Another difficulty the women faced with regard to the issue, according to the professionals, is that, in their social interpretation, sex, which is synonymous with pleasure, is inappropriate in a disease situation. The social interpretation that sex is only present in healthy life inhibits them from sharing these issues with the team.

Thinking about sex, sex is pleasure, it's joy, it's... I don't know. It's something good and, at that time, she doesn't have to focus on that. Because I actually think that they believe, like, that it would be a "sin" to address it at a time when they should be focusing on other things, right? (I13, nurse, Ward)

As sex is linked with the idea of pleasure, patients often feel guilty and ashamed to think about sex at such a time. They do not question health professionals about these issues because they think it is inappropriate^(^ 7 ^)^. *However, sexuality should be understood as a multi-faceted concept, including sexual function, issues relevant to sexual self-confidence and relationships* *(* 28 *)* *.*

The team also perceived that women experience their sexuality quite differently from men and are totally influenced by the circumstances. The negative burden associated with having a stigmatizing disease, such as cancer, influences their sex drive and their more admissible concerns involve life, children and how to pay the bills, etc.

Women take all problems to bed. We take everything, work problems, someone who gave you a nasty look... Now, imagine that you're thinking about what he's thinking, "where's your breast, 'cause you used to have two, but now you don't". How are you going to put on a negligee, go through with a whole sex game, how are you going to dress up... (I4, nurse, Outpatient clinic)

In the interpretation of the professionals, the women have difficulty disconnecting from the personal and family issues in their environment and are unable to think about sex naturally amidst so many adverse consequences originating from the disease and treatment process.

## Conclusion

This study allowed the identification of the barriers which hinder the exploration of the issue of sexuality in the nursing care of women with gynecological and breast cancer in the health institution where the study was developed. The actions of the nursing professionals studied reproduce the innumerable influences upon them, such as the values and beliefs present in society related to sexuality, precepts of the present-day health model, specific characteristics of the organization of the nursing work in the institution in question and characteristics of oncology care, among others.

It can be concluded that the systematic inclusion of the issue in nursing care routines requires changes in the health paradigm and the work dynamics, as well as reflection on personal values and the social interpretations related to the issue, aiming to contribute to the collective task of divesting sexuality of the taboos and prejudices which accompany it. In this way, sexuality can be seen as a dimension which is essential to human beings and to their relationships, even in situations of serious and potentially fatal diseases, such as cancer.

Furthermore, this study contributes to the scientific literature as it examines the experiences of Brazilian nursing professionals regarding this issue. The scientific knowledge produced up to this moment has neglected the importance of investigating the processes that lead nurses to avoid the issues of sexuality in care practices. Elucidating these barriers is an important step to sensitize professionals to the needs of the patients for integral healthcare in the oncology setting.

Further research is necessary, examining this topic from the patients' perspective, with the aim of confronting the perceptions related to addressing this issue in nursing care practices in the Brazilian context.
